# Efficacy of PermaNet® 3.0 and PermaNet® 2.0 nets against laboratory-reared and wild *Anopheles gambiae* sensu lato populations in northern Tanzania

**DOI:** 10.1186/s40249-016-0220-z

**Published:** 2017-01-18

**Authors:** Eliningaya J. Kweka, Lucile J. Lyaruu, Aneth M. Mahande

**Affiliations:** 1 Tropical Pesticides Research Institute, Division of Livestock and Human Diseases Vector Control, Mosquito Section, P.O. Box 3024, Arusha, Tanzania; 2 Department of Medical Parasitology and Entomology, Catholic University of Health and Allied Sciences, P.O. Box 1464, Mwanza, Tanzania; 3 Tropical Pesticides Research Institute, Division of Livestock and Human Diseases Vector Control, Mabogini field station, Moshi, Tanzania

**Keywords:** Exophily, Long-lasting insecticidal nets, *Anopheles gambiae*, Experimental hut, Mortality, Personal protection rate, Resistance, Tanzania

## Abstract

**Background:**

Mosquitoes have developed resistance against pyrethroids, the only class of insecticides approved for use on long-lasting insecticidal nets (LLINs). The present study sought to evaluate the efficacy of the pyrethroid synergist PermaNet® 3.0 LLIN versus the pyrethroid-only PermaNet® 2.0 LLIN, in an East African hut design in Lower Moshi, northern Tanzania. In this setting, resistance to pyrethroid insecticides has been identified in *Anopheles gambiae* mosquitoes.

**Methods:**

Standard World Health Organization bioefficacy evaluations were conducted in both laboratory and experimental huts. Experimental hut evaluations were conducted in an area where there was presence of a population of highly pyrethroid-resistant *An. arabiensis* mosquitoes. All nets used were subjected to cone bioassays and then to experimental hut trials. Mosquito mortality, blood-feeding inhibition and personal protection rate were compared between untreated nets, unwashed LLINs and LLINs that were washed 20 times.

**Results:**

Both washed and unwashed PermaNet® 2.0 and PermaNet® 3.0 LLINs had knockdown and mortality rates of 100% against a susceptible strain of *An. gambiae* sensu stricto*.* The adjusted mortality rate of the wild mosquito population after use of the unwashed PermaNet® 3.0 and PermaNet® 2.0 nets was found to be higher than after use of the washed PermaNet® 2.0 and PermaNet® 3.0 nets.

**Conclusions:**

Given the increasing incidence of pyrethroid resistance in *An. gambiae* mosquitoes in Tanzania, we recommend that consideration is given to its distribution in areas with pyrethroid-resistant malaria vectors within the framework of a national insecticide-resistance management plan.

**Electronic supplementary material:**

The online version of this article (doi:10.1186/s40249-016-0220-z) contains supplementary material, which is available to authorized users.

## Multilingual abstracts

Please see Additional file 1 for translations of the abstract into the five official working languages of the United Nations.

## Background

For the past three decades, significant progress in malaria control has been largely attributed to the widespread use of insecticide-based vector control interventions including indoor residual spraying (IRS) and long-lasting insecticidal nets (LLINs) [1–5]. A LLIN is a factory-treated mosquito net that is expected to retain its biological activity for a standard number of washes and a for a period of not less than 3 years but not more than 5 years [6]. Currently, a LLIN would be expected to retain its biological activity for at least 20 standard washes under laboratory conditions and 3 years of recommended use under field conditions, as defined in the recently-updated World Health Organization (WHO) guidelines [7].

The increasingly insecticide-resistant population of *Anopheles gambiae* sensu lato mosquitoes (hereafter referred to as *An. gambiae*) across Africa could represent a threat to the tools currently used for vector control [8–14]. Resistance to every currently used insecticide has been found and many factors are believed to increase vector resistance including the extensive use and misuse of the same classes of insecticides in agriculture and public health sectors [7].

Combined insecticides have reduced the level of resistance in the vector population [15], and rotating insecticides periodically has shown to be effective against wild vector populations or in delaying the build-up of insecticides resistance among vectors [11, 15, 16]. However, none of these options are able to reduce the metabolic activity of the mosquito against insecticides. Discovering a tool that can reduce or inhibit the enzymatic activity of the mosquitoes against classes of insecticides is a top priority to curb the resistance problem.

LLINs that use two unrelated insecticides or an insecticide plus a synergist have been shown to have increased efficacy against pyrethroid-resistant malaria vectors [17]. The incorporation of the synergist, piperonyl butoxide (PBO), in LLINs is able to significantly reduce or inhibit the enzymatic detoxification of insecticides, thus increasing the toxicity against mosquitoes [18]. PBO is an inhibitor of mixed-function oxidases implicated in pyrethroid resistance and also increases the rate of insecticide uptake through the mosquito cuticle [11, 16]. There are currently two pyrethroid synergist LLINs recommended by the WHO, namely, Olyset^®^ Plus and PermaNet^®^ 3.0 [19]. The latter is a combination of deltamethrin coated on the net’s polyester side panels and a mixture of deltamethrin and PBO on the polyethylene top panel.

In this study, we compared the pyrethroid synergist PermaNet^®^ 3.0 LLIN, the pyrethroid-only PermaNet® 2.0 LLIN and an untreated net, following standard WHO procedures [20]. This was done to determine the comparative efficacy against a free-flying, wild population of *An. gambiae* mosquitoes. As per recommended WHO standard experimental hut trials measurable outcomes, efficacy was measured in terms of blood-feeding inhibition, deterrence, induced exophily and mortality (both immediately and after 24 h).

## Methods

### Study site

The PermaNet test was conducted in Lower Moshi rice irrigation schemes in northern Tanzania using an East African experimental hut design. The experimental huts used in this trial were situated in Mabogini village, Moshi Rural District, northern Tanzania. They were constructed according to an East African experimental hut design first described elsewhere [17, 21]. The study area was chosen because of its high mosquito density throughout the year and a well-known status of insecticide resistance of the malaria vectors, *An. arabiensis.* Malaria vectors in this area are currently resistant to pyrethroids [14, 22, 23].

### Washing procedures

Before washing each LLIN, 20 g of *Persil Savon de Marseille* (Unilever) was added to 10 l of dechlorinated water and dissolved for 30 min. Each net was washed, immersed in the soap solution and manually agitated by hand protected with gloves for 10 min for an average of 20 rotations per minute. Nets were thereafter rinsed twice in dechlorinated tap water and dried in the shade. After been dried, the nets were stored in a dark room at ambient temperature. PermaNet® 2.0 and PermaNet® 3.0 LLINs were washed 20 times, while untreated nets were washed the same.

### Susceptibility test

A susceptibility test was conducted using the commonly-used pyrethroids, deltamethrin (0.05%) and permethrin (0.75%). Susceptibility tests were done following the procedures defined in the WHO Pesticide Evaluation Scheme (WHOPES) protocol. [20] Mosquitoes population was considered susceptible when mortality was between 98 and 100%. A mortality rate of less than 98% suggested the existence of a resistant population [20]. If the mortality rate was less than 90%, it indicated the existence of a resistance gene in the population against the evaluated insecticide [6, 20].

### Evaluated materials

Rectangular PermaNet® 2.0 and PermaNet® 3.0 LLINs were provided by their manufacturer, Vestergaard Frandsen SA, Denmark. Untreated nets were purchased from local shops; they were rectangular polyester nets (manufactured by A to Z Textile Mills, Arusha, Tanzania (http://www.azpfl.com/index.php/en/)), white in colour with no insecticide treatment. The PermaNet^®^ 2.0 was polyester and coated with 55 mg/m^2^ ± 25% deltamethrin. The PermaNet® 3.0 had a polyethylene roof with 2.8 g/kg ± 25% deltamethrin and 4.0 g/kg ± 25% PBO, and sides coated with 2.8 g/kg ± 25% deltamethrin. PBO is a synergist compound that elevates the rate of penetration of the insecticide into the insect cuticle [24] and inhibits the enzymatic ability of the insect to breakdown the insecticide [11].

Bioassays were conducted for all nets before and after washing. Bioassays were also conducted for nets that were washed 20 times before the experimental hut trial commenced and for all nets (washed and unwashed) after the experimental hut trial ended. The cone bioassays were executed for the roof, two long sides and two shots sides (legs and head positions of the nets). Five replicates were taken for each bioassay. All net samples were folded in aluminium foil and placed individually in a labelled clean black plastic bag prior to the assays being conducted.

### Bioassays on mosquito nets

The standard WHO method for cone bioassays was followed to determine the bioefficacy of LLINs against field-derived populations, permethrin-selected population and susceptible laboratory-reared *Anopheles gambiae* s.s. (Kisumu strain) [20]. The Kisumu colony was established at the Tropical Pesticides Research Institute (TPRI) in 1992. The colony is 100% susceptible to all approved WHOPES pesticides, which are tested frequently and confirmed every 6 months for susceptibility status using the standard WHO susceptibility test.

At the TPRI insectary, five unfed *An. gambiae* s.s. females were exposed for three minutes, removed and kept in holding paper cups provided with 10% sugar solution. The knockdown rate was recorded at 60 min post-exposure and the mortality rate after 24 h. Two cone tests were performed for each side of the net and for each mosquito population including for the laboratory-susceptible population; 250 mosquitoes of each of the five populations were tested for each net type. Mosquitoes exposed to untreated nets were used as controls and all results in control with mortality rates above 20% were discarded. Corrected mortality was applied when control mortality was above 5% using the Abbott’s formula.

### Experimental hut trial study design

The following five treatment arms were compared: (i) unwashed PermaNet® 2.0 (P2.0UN); (ii) PermaNet® 2.0 washed 20 times (P2.0WA); (iii) unwashed PermaNet® 3.0 (P3.0UN); (iv) PermaNet® 3.0 washed 20 times (P3.0WA); and (v) untreated polyester net (UTN). Each net was punctured with six (4 cm × 4 cm) holes to simulate a community-used worn net. The treatment arms were rotated five times through the huts using 5 by 5 Latin square design.

A treatment was assigned to a particular hut for five nights before being rotated to the next hut. In each hut, there was a male volunteer who gave consent to participate in the study before the trial began. Based on the treatment arms, five sleepers were randomly rotated during the five nights in five huts. Five sleepers were rotated through five huts on consecutive nights. Five nets were available per treatment arm and each net was tested on a consecutive week during the 5 weeknight rotations. At the end of each rotation, the huts were cleaned and aired for 1 day and the treatments moved to the next hut. White sheets were laid over the veranda and floors in the rooms to ease the collection of knocked-down mosquitoes. Each morning after dawn, mosquitoes were collected using aspirators from the floor, walls, veranda traps and inside the nets, scored as dead or alive and as fed or unfed, and identified to species using an Olympus BX41microscope (Olympus Corporation, Rochester, NY, USA). Live mosquitoes were kept for 24 h in paper cups with sugar solution to determine delayed mortality.

The main outcomes measured were: deterrence (defined as a reduction in hut entry relative to the control huts fitted with untreated nets); treatment-induced exophily (defined as the proportion of mosquitoes found in exit traps relative to the control huts); blood-feeding inhibition (defined as the proportional reduction in blood-feeding mosquitoes relative to untreated nets); and mortality (defined as the proportion of mosquitoes found dead).

The deterrence and blood-feeding inhibition of these outcomes are indicators of the personal protection rate, which can be estimated by the equation:

$$ \%\ \mathrm{Personal}\ \mathrm{protection}\ \mathrm{rate}=100\left({\mathrm{B}}_{\mathrm{u}}\hbox{--} {\mathrm{B}}_{\mathrm{t}}\right)/{\mathrm{B}}_{\mathrm{u}}, $$

where B_u =_ is the total number of blood-fed mosquitoes in the huts with untreated nets and B_t_ is the total number of blood-fed mosquitoes in the huts with treated nets.

The overall killing effect of the treatment was estimated by the equation:

$$ \mathrm{Insecticidal}\ \mathrm{effect}\;\left(\%\right)=100\left({\mathrm{K}}_{\mathrm{t}}\hbox{--} {\mathrm{K}}_{\mathrm{u}}\right)/{\mathrm{T}}_{\mathrm{u}}, $$

where K_t_ is the number of mosquitoes killed in the huts with treated nets, K_u_ is the number of mosquitoes found dying in the huts with untreated nets and T_u_ is the total number of mosquitoes collected from the huts with untreated nets.

The criteria for approval of PermaNet® 3.0 was that, the PermaNet^®^ 3.0 LLINs that were washed 20 times or more should perform equal to or better than a conventionally treated washed net just before exhaustion. Twenty washes are set by the WHO as the average number of washes a LLIN is likely to incur during its life, assuming nets are washed 4 times a year and last 3 to 5 years.

### Data analyses

For cone bioassays, knockdown and mortalities were compared for individual samples using regression analyses. Data, aggregated for mosquito population, net type and net section, were assessed using logistic regression for proportional data outcomes (proportions of blood-feeding and dying mosquitoes and those exiting the hut each night). All data for each net were then combined for net sections.

## Results

### Cone bioassay with susceptible mosquitoes

#### Before washing

**Fig. 1:**
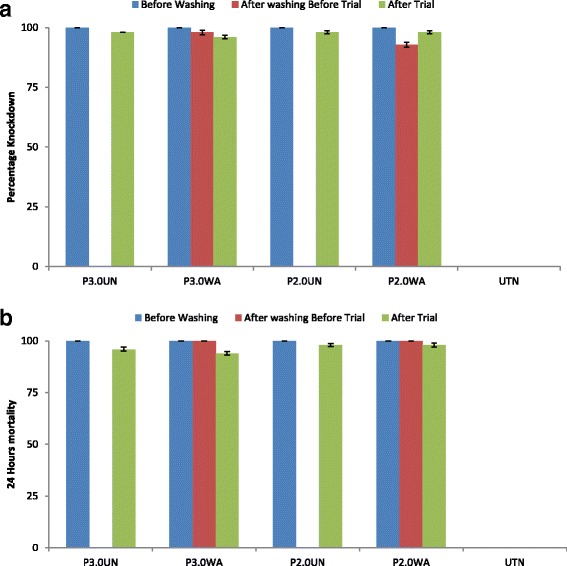
Contact bioassays for the detection susceptibility test for permethrin tolerant *An. gambiae* . **a** knockdown effect; **b** mortality rate after 24 h before washing, after washing 20 times and after experimental hut trial

The knockdown effect for the treated nets 60 min after exposure was 100%, while the mortality rate after 24 h was 100%. The untreated net knockdown effect and mortality rate was 0% (see Fig. 1a and b).

#### After 20 washes

After 20 washes, the knockdown effect varied between the nets: in untreated nets, it was 0.0%, in P3.0WA it was 98.0% and in P2.0WA it was 92.8%. The mortality rate after 24 h was 0%, 100% and 100% for the untreated net, P3.0WA and P2.0WA, respectively (see Fig. 1a and b).

#### After the experimental hut trial

The washed, unwashed and untreated nets showed variations in both the knockdown and mortality rates after the experimental hut trial. The knockdown effect 60 min after exposure was 0%, 100%, 98%, 98% and 96%, while the mortality rate after 24 h was 0%, 96%, 98%, 98% and 94% for UTN, P3.0UN, P2.0UN, P2.0WA and P3.0WA, respectively (see Fig. 1a and b).

### Cone bioassays with a resistant colony

#### Before washing

**Fig. 2:**
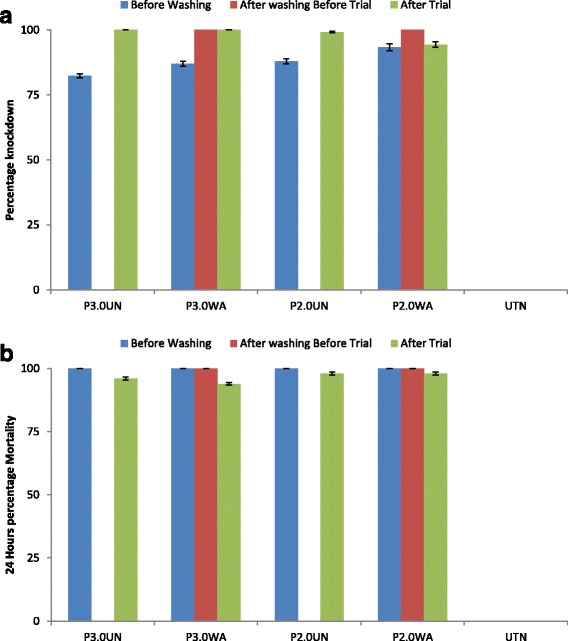
Contact bioassays for the permethrin tolerant *Anopheles gambiae,* **a** knockdown effect; **b** mortality rate after 24 h, before washing, after washing 20 times and after experimental hut trial

The knockdown effect 60 min after exposure and the mortality rate after 24 h for varied for unwashed treated nets (PermaNet brands) for a resistant population of *An. gambiae* (see Fig. 2a and b).

#### After 20 washes

The knockdown effect of nets washed 20 times varied. The knockdown effect was 0%, 100% and 100% after 60 min for UTN, P3.0WA and P2.0WA, respectively. The mortality rate after 24 h was 0%, 100% and 94.4% for UTN, P3.0WA and P2.0WA, respectively (see Fig. 2a and b).

#### After the experimental hut trial

The nets’ efficacy after the hut trial varied considerably. The knockdown effect 60 min after exposure was 0.0%, 100.0%, 100.0%, 99.2% and 94.4%, while the mortality rate after 24 h was 0.0%, 100.0%, 100.0%, 98.4% and 92.8% for UTN, P3.0UN, P3.0WA, P2.0UN and P2.0WA, respectively (see Fig. 2a and b).

### Deltamethrin-susceptibility test using wild and laboratory-reared mosquito populations

For the wild-caught adult female *An. gambiae* mosquitoes exposed to deltamethrin-treated WHO kit, the mortality rate was found to be 28.8%. Meanwhile, the survival rate was found to be 71.2% 24 h after exposure to WHOPES insecticide-treated paper. The mortality rate for the laboratory colony of *An. gambiae* s.s. was 100% against deltamethrin.

### Permethrin-susceptibility test using wild and laboratory-reared mosquito populations

For the adult female *An. gambiae* mosquitoes exposed to permethrin-treated WHO kit, the mortality rate was found to be 29.0%. Meanwhile, the survival rate was found to be 71.0% 24 h after exposure to WHOPES insecticide-treated paper. The mortality for *An. gambiae* s.s laboratory colony (control) was 100% against deltamethrin.

### Experimental hut trial

In the experimental hut trial, the efficacy of the evaluated nets was measured using the following parameters (see Table 1):

- ***Deterrence***: The rate of deterrence of mosquitoes was 78.7%, 78.7%, 80.0% and 86.7% for P2.0WA, P3.0UN, P3.0WA and P2.0UN, respectively.
- ***Exophily***: The number of mosquitoes found exiting the huts as the repellence effect of the LLINs treated nets varied from each due to different washing and brands. Exophily was found to be 9.3%, 90.0%, 93.8%, 81.3% and 80.0% for UTN, P2.0UN, P2.0WA, P3.0UN and P3.0WA, respectively.
- ***Blood-feeding inhibition***: Blood-feeding inhibition was found to be 100% for all treated nets as compared to the control.
- ***Mortality***: The corrected mortality due to mortality exceeded 5% in control for mosquitoes collected in the huts after 24 h was 59.5% for P2.0UN, 36.7% for P2.0WA, 49.3% for P3.0UN and 32.4% for P3.0WA.

**Table 1:** Evaluation of behavioural response in *An. gambiae* mosquitoes wild population during the experimental hut trial using five different treatments

Parameter	Summary data	Treatment (T)				
		UTN (u)	P2.0UN	P2.0WA	P3.0UN	P3.0WA
Deterrence	Total number of females caught	75	10	16	16	15
	Females caught/night	3	0.4	0.6	0.6	0.6
	Deterrence (%)		86.7	78.7	78.7	80.0
Exophily	Number of females in exit traps and veranda	7	9	15	13	12
	Exophily (%)	9.3	90.0	93.8	81.3	80.0
	95% confidence limits	4.8–12.9	86.3–96.1	89.9–97.2	74.8–87.9	77.2–83.3
Blood-feeding	Number of blood-fed females (B)	3	0	0	0	0
	% blood-fed	**4**	**0**	**0**	**0**	**0**
	95% confidence limits	1.5–6.8				
	Blood-feeding inhibition (%)		100	100	100	100
Mortality	Number of dead females in the morning (immediate mortality)	1	5	5	8	5
	Number of dead females after 24 h (delayed mortality)	0	1	1	0	0
	Total number of dead females (K)	1	6	6	8	5
	Overall mortality (%)	**1.3**	**50.0**	**31.3**	**50.0**	**33.3**
	95% confidence limits	0.6–2.1	44.6–54.9	27.7–35.6	46.5–56.2	27.6–49.3
	Mortality corrected for control (%)	-	59.5	36.7	49.3	32.4
SUMMARY	Personal protection rate (%)	-	100	100	100	100
	Killing effect rate (%)		50	50	70	40

### Personal protection rate and killing effect rate of nets

**Fig. 3:**
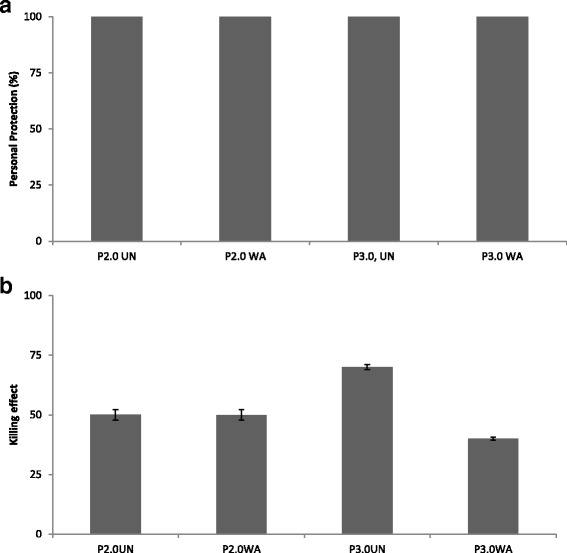
Personal protection rate (**a**) and killing effects (**b**) of evaluated nets against wild populations of *An. gambiae* mosquitoes

The personal protection efficiency of all nets was 100%, while the killing effect ranged between 40 and 70% among the various net treatments (see Fig. 3a and b, and Table 1).

### *An. gambiae* species composition

**Fig. 4:**
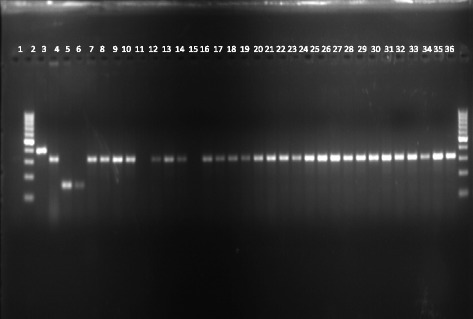
Species identification of wild *An. gambiae* mosquitoes. Lane 1 negative control, Lane 2 and 36 DNA ladder, Lane 3 *An. gambiae* positive control, Lane 4 *An. arabiensis* positive control, Lane 5 *An. quadriannulatus* positive control, Lane 6 *An. merus* positive control, Lane 7–35 DNA of mosquitoes

All identified specimens of *An. gambiae* s.l. were found to belong to the *An. arabiensis* species (see Fig. 4).

## Discussion

This study was conducted in Lower Moshi, in which a wild population of *An. gambiae* mosquitoes has been identified as having both phenotypic and metabolic resistance to insecticides [14, 22, 23, 25]. The study site has an *An. arabiensis* population. This scenario was previously reported by Ijumba and others in the early 1990s, when they found a composition of 95% *An. arabiensis* [26]. Another study conducted between 2010 and 2012 by Matowo and others found out of the 100% of mosquitoes in this area, 98% are of the *An. arabiensis* [23].

This study demonstrated that both unwashed LLINs and LLINs washed 20 times provided a high personal protection against *An. arabiensis* mosquitoes, which were found to be pyrethroid-tolerant according to the criteria provided by the WHO protocol for susceptibility test. [7] The nets were punctured to mimic community-used nets, but they still showed a great protection efficacy in spite of the holes. The personal protection rate for both unwashed LLINs and LLINs washed 20 times was found to be 100%. This is higher than what was reported in a previous study by Kitau and others, who tested intact nets [27]. This suggests that in an area where there is a population of resistant vectors, a person can be protected against mosquitoes if positioned under a bed net, but is vulnerable when outside the bed net [28]. Alternative personal protection tools other than bed nets, such as repellents, should be used for improved protection [4].

The differences in mortality rates might be attributed to phenotypic, knockdown resistance (kdr) or biochemical resistance mechanisms, as the mortality rates observed were very low [14, 22, 23, 29]. Similar scenario of low mortality was observed in areas with P450 and kdr resistance mechanisms, which have hampered the resistance rate among malaria vector control including *An. funestus* in South Africa where deltamethrin has been used intensively for IRS [30, 31]. In Cameroon, it was found that an evaluation of P450 activity in *An. gambiae* mosquitoes reduces the bioefficacy of permethrin-treated nets conducted in the laboratory [32, 33]. A combination of resistance mechanisms might be a major blocking factor to the control of malaria vectors by impairing the efficacy of combined insecticides or synergist such as PBO with insecticide [34]. In the study area, the predominant mechanisms are phenotypic and metabolic [22, 23, 35].

Both PermaNet® 2.0 (P2.0WA) and PermaNet® 3.0 (P3.0UN) were found to have lowest deterrence effects (78.0%) while P2.0UN had highest deterrence (86.7%). The washing effect could not be seen in terms of decrease of deterrence effect in PermaNet 3.0 but in PermaNet 2.0. However, the reduction in the mosquito numbers entering the hut or house increases the possibility of personal protection, but may not be a reliable indicator of LLIN efficacy as sometimes variation in protection efficacy have been observed with similar nets [13]. In Benin, the protection efficiency of an insecticide-treated net was found to be reduced to 50% in an area with a resistant population of *An. gambiae* mosquitoes, while in susceptible areas it was 100% [36]. These findings indicate that PermaNet® 2.0 and improved PermaNet® 3.0 (with PBO) are advanced tools for protection against resistant populations of *An. gambiae* mosquitoes [8, 17, 37]. This efficacy has also been observed in Ethiopia [9] and Cote d’Ivoire [18]. A similar study conducted in Muheza, Tanzania found that the personal protection rates for PermaNet® 3.0 and PermaNet® 2.0 washed 20 times were 71 and 73%, respectively [17]. The cause of these variations in the personal protection rate between and within the two PermaNet brands done in Tanzania are still not well understood. But it has been suggested that they are attributed to the differences in insecticide resistance among wild mosquito populations and may be their different resistance mechanisms involved [17, 23, 29].

The mortality rates after use of either the washed or unwashed PermaNet® 2.0 and PermaNet® 3.0 LLINs were found to be between 32.4 and 59.5%. Low mortality rates have been recorded with deltamethrin-treated nets elsewhere including in Cote d’Ivoire, Southern Benin and Burkina Faso, which are all areas with mosquito populations that have a reduced susceptibility to permethrin and deltamethrin; mortality was below 40% in all those three areas mentioned above [31, 36]. The incorporation of deltamethrin and PBO in nets has been found to improve the mortality rates of mosquitoes in areas with highly-resistant populations due to the synergistic effect [38–40]. PBO has been found to increase the cuticle penetration rate of insecticides, hence increasing the mortality rate of the targeted species by increasing insecticide toxicity [22, 41]. It has been reported that deterrence varies widely from 0 to 70% during huts rotation for similar LLINs against wild resistant *An. gambiae* populations [17].

Natural exophily was found to be 9.3% for untreated nets, while it was >80.0% for the treated washed and unwashed PermaNet® 2.0 and PermaNet® 3.0 LLINs. Despite of resistance level of wild population of *An. arabiensis* against deltamethrin still induced exophily by both brands of PermaNet either washed or unwashed was higher and blood-feeding inhabitation was 100%. The deltamethrin resistance level has increased significantly in the past 10 years in Lower Moshi [14, 22, 23, 35].

Although the nets were holed to mimic community-used nets, the PermaNet® 3.0 and 2.0 still had irritancy effect to hold off *An. arabiensis* not feeding on volunteer under the bed net In Cote d’Ivoire, a resistant wild population of *An. gambiae* mosquitoes was found to have low feeding succession rates, but it might have been due to intact (not holed) nets being utilised [18].

## Conclusions

The present study reveals that the use of both unwashed and washed PermaNet® 2.0 LLINs was associated with higher mortality rates than with the PermaNet® 3.0 LLINs. Exophily and deterrence rates were similar. A community-based evaluation of PermaNet® 3.0 and PermaNet® 2.0 LLINs in an area with a similar level or a higher phenotypic and metabolic resistance in mosquitoes will allow for comparable results and thus for a better conclusion.

The observed impact of the unwashed PermaNet® 3.0 LLIN compared to the unwashed PermaNet® 2.0 LLIN was confirmed to be higher in terms of the killing effect (70% versus 50%, respectively). Similar results were obtained for the washed PermaNet 2.0® and PermaNet® 3.0. This low killing effect was associated with an elevated resistance to pyrethroid among the wild mosquito population [25, 42]. The highest deterrence effect, personal protection and feeding inhibition was the most outstanding factor to advocates these nets use in areas with elevated insecticides resistance.

## Additional file

Additional file 1: Multilingual abstracts in the five official working languages of the United Nations. (PDF 683 kb)

## Abbreviations

IRS: Indoor residual spray
kdr: knockdown resistance
LLIN: Long-lasting insecticidal net
P2.0UN: PermaNet® 2.0 unwashed net
P2.0WA: PermaNet® 2.0 net washed 20 times
P3.0UN: PermaNet® 3.0 unwashed net
P3.0WA: PermaNet® 3.0 net washed 20 times
PBO: Piperonyl butoxide
TPRI: Tropical Pesticides Research Institute
UTN: Untreated net
WHO: World Health Organization
WHOPES: WHO Pesticide Evaluation Scheme
