# Supplementary material for: Efficacy of PermaNet® 3.0 and PermaNet® 2.0 nets against laboratory-reared and wild Anopheles gambiae sensu lato populations in northern Tanzania
Source: Infect Dis Poverty. 2017 Jan 18;6:11. doi: 10.1186/s40249-016-0220-z (PMC5242039; doi:10.1186/s40249-016-0220-z)
Supplement: Additional file 1: — Multilingual abstracts in the five official working languages of the United Nations. (PDF 683 kb) [file 40249_2016_220_MOESM1_ESM.pdf]

Translation of the abstract into the five official working languages of the United Nations

فعالية برمانيت 3.0 وبرمانيت 2.0 ضد تجمعات الأنوفيلة الغامبية الناشئة في المعامل والبرية منها في شمال تنزانيا

ايليمينجاوا جي كواكا، لوسيل جي ليارو، آنيس ام ماهاندي

ملخص

الخلفية: قام البعوض بتطوير مقاومته ضد البيريثرويد، صنف المبيدات الحشرية الوحيد الموافق على استخدامه ضد الناموسيات المعالجة بمبيدات مديدة المفعول. سعت الدراسة الحالية لتقييم فعالية البيريثرويد المحفز برمانيت 3.0 في مواجهة البيريثرويد العادي برمانيت 2.0، في الغرب الإفريقي وفي منطقة أكواخ تجريبية في موشي السفلى في شمال تنزانيا. في هذا الإطار، تم الكشف عن مقاومة بعوض الأنوفيلية الغامبية لمبيدات البيريثرويد الحشرية.

الطرائق: تقييمات الفعالية البيولوجية لمنظمة الصحة العالمية التي تمت في المختبر أو في الأكواخ التجريبية. اختبارات الأكواخ التجريبية التي تمت في منطقة تحتوي على تجمعات لبعوض الأنوفليس أرابينسيس لديها مقاومة شديدة للبيرثرويد. جميع منتجات النيتس التي تم استخدامها كان موضوعاً لتجارب بيولوجية ومن ثم لتجارب الأكواخ التجريبية. وقد تمت المقارنة بين موت البعوض وتثبيط تغذية البعوض على الدم ومعدلات الحماية الشخصية في حالات استخدام منتجات نيتس غير المعالجة والتي لم يتم غسلها والناموسيات المُعالجة بمبيدات مديدة المفعول التي لم يتم غسلها والناموسيات المُعالجة بمبيدات مديدة المفعول التي تم غسلها 20 مرة.

النتائج: كل من منتجي برمانيت 2.0 الذي تم غسله والذي لم يتم غسله وبرمانيت 3.0 ضد الناموسيات المعالجة بمبيدات مديدة المفعول حيث تم القضاء عليها ومعدل موتها هو 100% ضد السلالة المعرضة لنقل المرض. تبين أن معدل الوفاة القياسي لتجمعات البعوض المتوحش بعد استخدام منتجي برمانيت 3.0 الذي لم يتم غسله وبرمانيت 2.0 هي أكبر بعد استخدام برمانيت 2.0 الذي تم غسله وبرمانيت 3.0.

خلاصات: بالنظر إلى ازدياد مقاومة بعوض الأنوفيلية الغامبية للبيريثرويد في تنزانيا. فإننا ننصح بأن يولى الاهتمام في توزيعها في مناطق تنتشر فيها نواقل الملاريا المقاومة للبيريثرويد في إطار الخطة الوطنية للمبيدات الحشرية المقاومة.

Translated from English version into Arabic by sjaatoul, through

TRANSLATORS WITHOUT BORDERS

PermaNet® 3.0 和 PermaNet® 2.0 长效蚊帐对坦桑尼亚北部地区实验室饲养和野生冈比亚按蚊复合体的防护效果

Eliningaya J. Kweka, Lucile J. Lyaruu, Aneth M. Mahande

摘要

引言：蚊媒已对唯一批准应用于长效杀虫剂处理蚊帐（LLINs）的杀虫剂---拟除虫菊酯产生抗性。本研究的目的是在坦桑尼亚北部 Lower Moshi 进行实验小屋设计以评估拟除虫菊酯增效剂 PermaNet® 3.0 长效蚊帐和单拟除虫菊酯 PermaNet® 2.0 长效蚊帐的防护效果。在此条件下，确定冈比亚按蚊（*Anopheles gambiae*）已对拟除虫菊酯产生抗性。

方法：采用世界卫生组织标准生物效能评估方法在实验室和实验小屋条件下进行测试。在对拟除虫菊酯具有高度抗性的阿拉伯按蚊群体进行实验小屋评估。所有长效蚊帐均经锥形生物测试后用于实验小屋试验。并比较未经处理的蚊帐、未洗涤的 LLINs 和洗涤 20 次的 LLINs 三组间按蚊死亡率、吸血抑制和个人防护率的差异。

结果：洗涤和未洗涤的 PermaNet® 2.0 和 PermaNet® 3.0 长效蚊帐对冈比亚按蚊复合体（*An. gambiae* sensu stricto）非抗性株均有 100%的击倒率和致死率。与洗涤组相比，未洗涤组的野生群体的校正死亡率较高。
结论：鉴于坦桑尼亚冈比亚按蚊对拟除虫菊酯的抗性日益增加，我们建议在国家杀虫剂耐药性管理计划的框架下特别关注那些有拟除虫菊酯抗性按蚊分布的地区。

Translated from English version into Chinese by Xin-Yu Feng, edited by Pin Yang， through

CDC
CHINESE CENTER FOR DISEASE CONTROL AND PREVENTION
NATIONAL INSTITUTE OF PARASITIC DISEASES

Efficacité de PermaNet® 3.0 et PermaNet® 2.0 moustiquaires contre les populations sensu lato d'*Anopheles Gambiae* sauvages et élevées en laboratoire, au Nord de la Tanzanie

Eliningaya J. Kweka, Lucile J. Lyaruu, Aneth M. Mahande

RESUME
Contexte: Les moustiques ont développé une résistance aux pyréthroïdes, seule classe d'insecticides approuvés pour être utilisés sur les Moustiquaires Imprégnées à Efficacité Durable (MIED). La présente étude a cherché à évaluer l'efficacité de la MIED à synergiste pyréthroïde PermaNet® 3.0 contre la MIED à pyréthroïde seul PermaNet® 2.0, dans une case expérimentale d'Afrique de l'Est au Moshi Inférieur, au Nord de la Tanzanie. Dans ce cadre, la résistance aux insecticides pyréthroïdes a été déterminée chez les moustiques *Anopheles Gambiae*.
Méthodes: les évaluations des normes de bio-efficacité de l'Organisation Mondiale de la Santé ont été effectuées aussi bien en laboratoire que dans les cases expérimentales. Les évaluations de la case expérimentale ont été effectuées dans une région où il y avait la présence d'une population de moustiques *An. Arabiensis* hautement résistants aux pyréthroïdes. Toutes les moustiquaires utilisées ont été soumises à des bio-essais en cône puis aux essais sur les cases expérimentales. Le taux de mortalité des moustiques, l'inhibition de l'alimentation en sang et la protection personnelle ont été comparés entre les moustiquaires non traitées, les MIED non lavées et les MIED ayant été lavées 20 fois.
Résultats: Les MIED PermaNet® 2.0 et PermaNet® 3.0 lavées et non lavées ont eu des taux d'action efficace et de mortalité de 100% sur une souche sensible d'*An. Gambiae* stricto sensu. Le taux de mortalité ajusté de la population de moustiques sauvages après utilisation de moustiquaires PermaNet® 3.0 et PermaNet® 2.0 non lavées s'est avéré être plus élevé qu'après l'utilisation de moustiquaires PermaNet® 2.0 et PermaNet® 3.0 lavées.
Conclusions: Etant donné l'incidence croissante de la résistance aux pyréthroïdes des moustiques *An. Gambiae* en Tanzanie, nous recommandons de prendre en considération leur distribution dans les régions à vecteurs de paludisme résistant aux pyréthroïdes dans le cadre du plan national de gestion de la résistance aux insecticides.

Translated from English version into French by Ode Laforge, through

Эффективность воздействия сеток PermaNet® 3.0 и PermaNet® 2.0 на популяции разводимых в лаборатории и диких комаров *Anopheles gambiae sensu lato* в Северной Танзании

Элинингая Дж. Квека (Eliningaya J. Kweka), Люсиль Дж. Лярю (Lucile J. Lyaruu), Анеф М. Маханде (Aneth M. Mahande)

АННОТАЦИЯ

Краткое описание. У комаров выработалась устойчивость к пиретроидам - единственному классу инсектицидов, одобренному для обработки противомоскитных сеток длительного действия (СОИДД). Данное исследование было проведено для оценки эффективности сеток PermaNet® 3.0 с пиретроидом-синергистом по сравнению с сетками PermaNet® 2.0, содержащими только пиретроид, в хижинах восточноафриканского типа в Нижнем Моши (Lower Moshi), Северная Танзания. В этой среде была выявлена устойчивость комаров *Anopheles gambiae* к пиретроидным инсектицидам.

Методы. Были сделаны стандартные оценки биоэффективности Всемирной организации здравоохранения в лабораторных и экспериментальных хижинах. Оценки в экспериментальных хижинах были проведены в районе, где присутствует популяция высокоустойчивых к пиретроидам комаров *An. arabiensis*. Все использованные сетки были подвергнуты коническому биоанализу и затем исследованию в экспериментальных хижинах. Были сравнены смертность комаров, подавление кровососущих свойств и степень защищённости людей для необработанных сеток, нестираных сеток СОИДД и сеток СОИДД, подвергнутых стирке 20 раз.

Результаты. Стираные и нестираные сетки PermaNet® 2.0 и PermaNet® 3.0 показали 100% убойное воздействие и смертность у восприимчивого штамма *An. gambiae sensu stricto*. Скорректированный показатель смертности популяции диких комаров после применения нестираных сеток PermaNet® 3.0 и PermaNet® 2.0 оказался выше, чем после применения стираных сеток PermaNet® 2.0 и PermaNet® 3.0.

Заключение. Учитывая рост устойчивости к пиретроидам комаров *An. gambiae* в Танзании, мы рекомендуем проанализировать его распределение в областях распространения устойчивых к пиретроидам переносчиков малярии в рамках национального плана управления борьбой с устойчивостью к инсектицидам.

Translated from English version into Russian by Natalia Potashnik, through

Eficacia de PermaNet® 3.0 y PermaNet® 2.0 contra complejos de *Anopheles gambiae* criados en laboratorio y silvestres sensu lato en el norte de Tanzania

Eliningaya J. Kweka, Lucile J. Lyaruu, Aneth M. Mahande

RESUMEN

Contexto: Los mosquitos han desarrollado resistencia contra los piretroides, la única clase de insecticidas aprobados para su uso en mosquiteros con insecticida de larga duración (LLINs, por sus siglas en inglés). El presente estudio evalúa la eficacia del piretroide sinergista PermaNet ® 3.0 LLIN versus el piretroide único PermaNet ® 2.0 LLIN, en una cabaña del África oriental en Lower Moshi, en el norte de Tanzania. En este contexto, se ha identificado la resistencia a insecticidas piretroides en los mosquitos *Anopheles gambiae*.

Métodos: Se realizaron evaluaciones estándar de la Organización Mundial de la Salud sobre la bioeficacia tanto en laboratorio como en cabaña experimental. Las evaluaciones de cabaña experimental se llevaron a cabo en una zona donde había presencia de una población de mosquitos altamente resistentes a los piretroides *An. arabiensis*. Todos los mosquiteros utilizados fueron sometidos a bioensayos de cono y luego a pruebas de cabaña experimental. La mortalidad de los mosquitos, la restricción a la alimentación de sangre y los ratios de protección personal se compararon entre mosquiteros no tratados, LLIN no lavados y LLIN lavados 20 veces.

Resultados Tanto el LLIN PermaNet® 2.0 como el PermaNet® 3.0 obtuvieron un efecto fulminante con tasas de mortalidad del 100 % contra una cepa susceptible de *An.. gambiae* sensu stricto. La tasa de mortalidad ajustada de la población de mosquitos silvestres después de usar los mosquiteros sin lavar PermaNet ® 3.0 y PermaNet ® 2.0 era más alta que después de usar los mosquiteros lavados PermaNet ® 2.0 y PermaNet ® 3.0.

Conclusiones: Dada la creciente incidencia de la resistencia a los piretroides en mosquitos *An. gambiae* en Tanzania, recomendamos que se preste atención a su distribución en zonas con vectores de piretroides resistentes a la malaria en el marco de un plan nacional de gestión de la resistencia a los insecticidas.

Translated from English version into Spanish by Maria Alejandra Aguada, through

TRANSLATORS
WITHOUT BORDERS
